# The Histone Variant H2A.Z C-Terminal Domain Has Locus-Specific Differential Effects on H2A.Z Occupancy and Nucleosome Localization

**DOI:** 10.1128/spectrum.02550-22

**Published:** 2023-02-23

**Authors:** Hannah Neumann, Celia Jeronimo, Jean-François Lucier, Emeline Pasquier, François Robert, Raymund J. Wellinger, Luc Gaudreau

**Affiliations:** a Department of Biology, Université de Sherbrooke, Sherbrooke, Quebec, Canada; b Montreal Clinical Research Institute, Montréal, Quebec, Canada; c Department of Medicine, Université de Montréal, Montréal, Quebec, Canada; d Center for Computational Science, Université de Sherbrooke, Sherbrooke, Quebec, Canada; e Department of Microbiology and Infectious Diseases, Université de Sherbrooke, Sherbrooke, Quebec, Canada; University of Montreal

**Keywords:** gene expression, H2A.Z, Swr1, yeast

## Abstract

The incorporation of histone variant H2A.Z into nucleosomes creates specialized chromatin domains that regulate DNA-templated processes, such as gene transcription. In Saccharomyces cerevisiae, the diverging H2A.Z C terminus is thought to provide the H2A.Z exclusive functions. To elucidate the roles of this H2A.Z C terminus genome-wide, we used derivatives in which the C terminus was replaced with the corresponding region of H2A (ZA protein), or the H2A region plus a transcriptional activating peptide (ZA-rII′), with the intent of regenerating the H2A.Z-dependent regulation globally. The distribution of these H2A.Z derivatives indicates that the H2A.Z C-terminal region is crucial for both maintaining the occupation level of H2A.Z and the proper positioning of targeted nucleosomes. Interestingly, the specific contribution on incorporation efficiency versus nucleosome positioning varies enormously depending on the locus analyzed. Specifically, the role of H2A.Z in global transcription regulation relies on its C-terminal region. Remarkably, however, this mostly involves genes without a H2A.Z nucleosome in the promoter. Lastly, we demonstrate that the main chaperone complex which deposits H2A.Z to gene regulatory region (SWR1-C) is necessary to localize all H2A.Z derivatives at their specific loci, indicating that the differential association of these derivatives is not due to impaired interaction with SWR1-C.

**IMPORTANCE** We provide evidence that the Saccharomyces cerevisiae C-terminal region of histone variant H2A.Z can mediate its special function in performing gene regulation by interacting with effector proteins and chaperones. These functional interactions allow H2A.Z not only to incorporate to very specific gene regulatory regions, but also to facilitate the gene expression process. To achieve this, we used a chimeric protein which lacks the native H2A.Z C-terminal region but contains an acidic activating region, a module that is known to interact with components of chromatin-remodeling entities and/or transcription modulators. We reasoned that because this activating region can fulfill the role of the H2A.Z C-terminal region, at least in part, the role of the latter would be to interact with these activating region targets.

## INTRODUCTION

The eukaryotic genome is organized as chromatin, of which the nucleosome is the basic unit. An individual nucleosome consists of an octameric histone core, formed by two H2A-H2B dimers and a H3-H4 tetramer, enveloped by 146 bp of DNA. This complex chromatin structure represents an obstacle to DNA-based processes, including gene transcription, DNA replication, and DNA repair. Therefore, for all of these transactions, nucleosomes must be remodeled in order to make DNA accessible. Several fundamental mechanisms can alter chromatin structure, including post-translational modifications of histones, which may modify their properties or interacting partners; ATP-dependent chromatin remodeling; and the replacement of replicative histones by histone variants which change the histone composition of nucleosomes. The latter mechanism creates specialized chromatin domains that can be permissive or not permissive to transcription (1).

H2A.Z, a histone variant of H2A, can be incorporated into nucleosomes in order to create such a specialized chromatin domain. H2A.Z is conserved among diverse eukaryotic organisms, from yeast to mammals, and replaces replicative H2A histone in 5% to 10% of all nucleosomes (2). In Saccharomyces cerevisiae, H2A.Z plays roles in the regulation of gene expression, DNA repair, maintenance of heterochromatic-euchromatic boundaries, chromosome segregation, and resistance to genotoxic stress (3–7). In general, the three-dimensional structure of H2A.Z-containing nucleosomes is similar to that of H2A-containing nucleosomes. However, there are subtle differences in the specific regions that differentiate structures of nucleosomes containing H2A versus H2A.Z, and this may explain their functional differences (8). The main divergence between these structures resides in the C-terminal docking domain of H2A.Z, which shows less than 40% sequence identity with the corresponding region of H2A.

In yeast, H2A.Z-nucleosomes are found on most gene promoters (9–16), and this genome-wide localization is conserved in *Drosophila*, chicken, plants and mammals (17–22). H2A.Z is almost exclusively incorporated in the +1 nucleosome in the direction of transcription (16, 23). Several studies have reported H2A.Z-dependent positive and negative regulation of gene transcription (3–6). It is thought that these specific H2A.Z nucleosomes regulate transcription by their particular positioning (9), by being less stable (12), and/or by contacting specific components of the transcription machinery (5). The acidic surface on H2A.Z-containing nucleosomes may constitute a binding platform for interacting partners such as chromatin-remodeling complexes or transcription coactivators (8). Moreover, H2A.Z is predominantly found in the promoters of inactive or weakly transcribed genes, and the H2A.Z-containing nucleosomes are thought to prepare these promoter areas to be disassembled for transcriptional initiation. Indeed, the assembly of the transcription pre-initiation complex (PIC) has been proposed to evict H2A.Z from gene promoters (24). More recent evidence suggests that H2A.Z eviction is dependent on RNA polymerase II and the Kin28/Cdk7 kinase, which phosphorylates serine 5 on the carboxy-terminal domain of the Pol II subunit, Rpb1 (25). Indeed, the association of H2A.Z with gene promoters is gradually lost following the induction of transcription in yeast and in mammals (4, 5, 9, 26). During transcription elongation, nucleosomes in gene bodies are also remodeled to allow the passage of RNA polymerase II. Finally, there is evidence that H2A.Z eviction from gene bodies is also performed by histone chaperones, such as FACT and Spt6 (27, 28).

In addition to RNA polymerase II-promoters, previous analyses of genome-wide location studies of H2A.Z have reported the presence of H2A.Z at a variety of genomic loci, including transcribed sequences (tRNAs, small nucleolar RNAs, rRNAs, and Ty elements) (9, 12, 14) and non-transcribed elements (the mating-type silent cassette, HZADs [Htz1-activated domains], telomeric regions, centromeres, replication origins, and double-stranded DNA breaks) (9–12, 14, 29–32). However, we note that there are also conflicting results on subsets of these localizations (10, 12). Although the role of H2A.Z in the maintenance of heterochromatic-euchromatic boundaries, chromosome segregation and DNA repair, have been investigated, the recruitment mechanisms of H2A.Z to the various genomic loci are still unclear, and the function of the H2A.Z C-terminal docking domain in these processes remains to be investigated.

Previous studies of the H2A.Z C-terminal region used either a complete replacement of the region by the corresponding region of H2A, H2A.Z C-terminal truncations of various lengths, or point mutations in the H2A.Z docking domain (5, 22, 33–37). Given that this terminal region is the one with most divergence from H2A, the results unsurprisingly showed that the H2A.Z C-terminal region is important for all functions associated with H2A.Z-containing nucleosomes (5, 22, 33–39).

**FIG 1:**
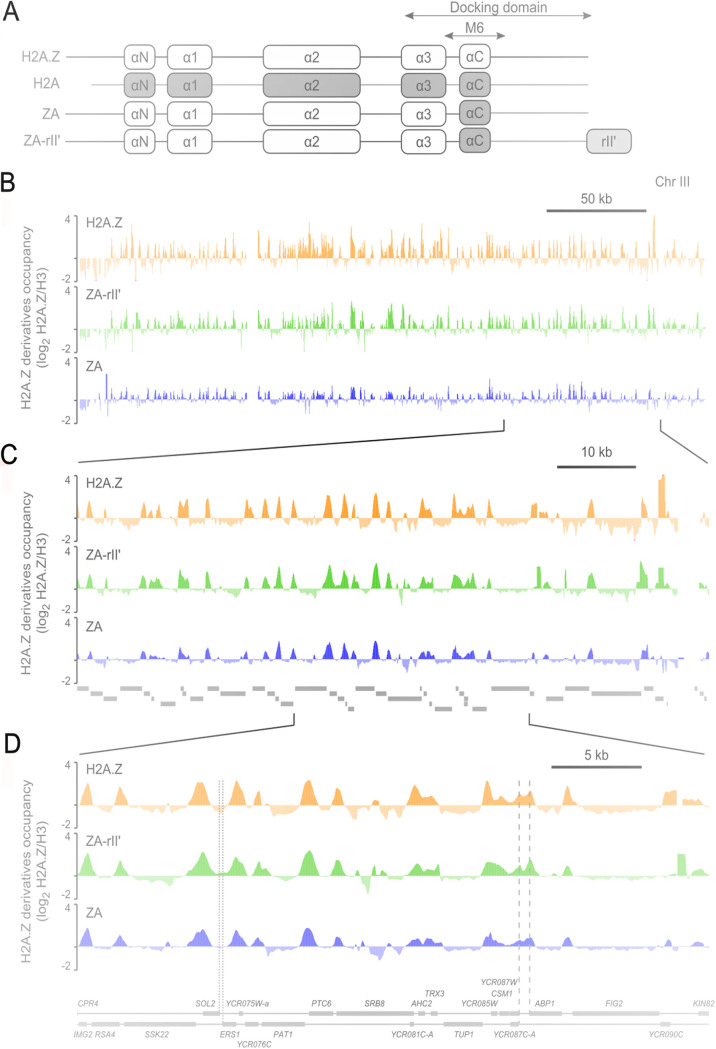
Genome-wide location analysis of H2A.Z, ZA, and ZA-rII′; zoom in on chromosome III. (A) Schematic representation of the H2A.Z derivatives used in this study. The fusions bear a hemagglutinin (HA) tag at their N terminus. The positions of the alpha helices, the M6 region, and the docking domain are indicated. (B) Log_2_ ratios of H2A.Z (orange), ZA-rII′ (green), and ZA (blue), normalized against histone H3, along chromosome III. (C) Zoom in on an 80-kb region between positions 215,000 and 295,000. Genes in this region are depicted in gray. (D) Zoom in on a 35-kb region between positions 240,000 and 275,000. Dotted lines indicate the 3′ region of two convergently transcribed genes (*SOL2* and *ERS1*) where no H2A.Z peak can be found. Dashed lines indicate the 5′ regions of two divergently transcribed genes (*YCR087C-A* and *ABP1*) where two separate H2A.Z peaks are observed.

Notwithstanding these studies, the involvement of the H2A.Z C-terminal docking domain has not been studied with regard to genome-wide localization and transcriptional control. Thus, in order to determine the role of the H2A.Z C-terminal docking domain in the functions of H2A.Z-containing nucleosomes, we used chimeric proteins derived from H2A.Z which bore modified C-terminal regions (Fig. 1A). Surprisingly, the results showed that the genome-wide localization of H2A.Z at many gene promoters is not dependent on its C-terminal region. However, modifications of the H2A.Z C-terminal docking domain leads to a loss of occupancy at these promoters and a slight shift of the position of the H2A.Z variant nucleosome toward the +2 nucleosome. However, the C terminus is required for correct positioning of H2A.Z nucleosomes near origins of replication, centromeres and promoters of snoRNA genes. The data also demonstrate that the special function of H2A.Z in regulation of the global transcriptome is dependent on its C-terminal region. Remarkably, a modification of the H2A.Z C terminus mostly affects the expression of genes lacking a H2A.Z nucleosome in the promoter, and several biological processes are affected. Therefore, our study also provides evidence for a global role of the H2A.Z C-terminal docking domain in regulation of gene transcription by coupling specific occupancy at gene promoters with the transcriptional outcome.

## RESULTS

### A modification of the C-terminal domain of H2A.Z affects its localization to certain loci and reduces its nucleosomal incorporation genome-wide.

Previous reports have shown that the H2A.Z C-terminal docking domain is essential for the incorporation of H2A.Z within chromatin and the role of H2A.Z in gene expression on a limited number of loci, but the necessity of this region regarding its genome-wide location and gene regulation has not been documented. To study the functional role of the H2A.Z C-terminal docking domain, we wanted to test whether replacing it with a transcriptional activating region could fulfill the same role. In fact, such H2A.Z chimeras have been used in the past, namely, the ZA and ZA-rII′ fusion proteins (5, 33). Briefly, the ZA fusion protein was obtained by replacing the C-terminal region of H2A.Z, which includes the M6 region (amino acids 97 to 134), with the corresponding region of H2A (amino acids 91 to 132) (Fig. 1A). Larochelle and Gaudreau (33) hypothesized that the H2A.Z C-terminal region could harbor a function reminiscent of a transcriptional activating region and therefore created a chimeric protein named ZA-rII′ by fusing the ZA protein to the Gal4 acidic transcriptional activating region (amino acids 840 to 881) (Fig. 1A). We reasoned that this transcriptional activating domain could, at least to a certain degree, mimic the function of the H2A.Z C-terminal docking domain and therefore complement the transcriptional phenotypes of Δ*htz1* cells. Previous data showed that the expression of the ZA chimera protein affects the expression of H2A.Z-regulated genes, namely, *GAL1*, *GAL7*, *GAL10*, and *PUR5*, and renders the cell more sensitive to genotoxic stress compared to H2A.Z-expressing cells (5, 33). Interestingly, the expression of the ZA-rII′ fusion protein allowed us to partially rescue the activation of the genes described above and the resistance to genotoxic stressors (33). Thus, these H2A.Z derivatives are a useful tool in order to dissect and study the functional role of the H2A.Z C-terminal region.

We set up a genome-wide localization assay (chromatin immunoprecipitation [ChIP] with DNA microarray, ChIP-chip) to map H2A.Z and the H2A.Z derivatives across the yeast genome. ChIP assays were performed using yeast strains bearing hemagglutinin (HA) epitopes on H2A.Z, ZA, and ZA-rII′. In each case, a ChIP for histone H3 (using an anti-H3 antibody) was hybridized together with the H2A.Z, ZA, or ZA-rII′ ChIPs to control for nucleosomal density. Genomic data visualization using the UCSC Genome Browser allowed us to determine that the H2A.Z, ZA, and ZA-rII′ proteins are distributed across the yeast genome in a non-random manner (see Fig. 1B for a map of chromosome III). The wild-type (WT) H2A.Z genome-wide location data obtained here agree and overlap very strongly with previously obtained localization results (6, 9–13, 15, 16, 36). In general, the ZA and ZA-rII′ peaks overlap the H2A.Z enrichment peaks, suggesting that, globally, all three proteins follow a similar non-random genomic distribution (Fig. 1B). Comparison of the localization signals revealed that the signal and enrichment peaks are diminished for the ZA fusion protein compared to H2A.Z and ZA-rII′ (Fig. 1C and D), suggesting at first sight that modification of the H2A.Z C-terminal docking domain does not grossly affect the localization of the enrichment peaks, but rather the level of incorporation of this protein within chromatin. To better appreciate this difference in the binding levels of the H2A.Z derivatives, we demonstrated binding enrichment relative to the transcription start site (TSS) of average genes, normalized either to H3 (Fig. S1A) or to input (Fig. S1B). A closer inspection of our genomic data suggests that H2A.Z is predominantly localized within promoter regions, as expected, and that the distributions of ZA and ZA-rII′ are also similar in that aspect (Fig. 1C and D). An enrichment of H2A.Z, ZA, and ZA-rII′ was observed upstream of every open reading frame (ORF), as shown in Fig. 1D, and very few genes show an enrichment of H2A.Z, ZA, and ZA-rII′ in the coding region. Intergenic regions where two genes converge, for example, *SOL2* and *ERS1* (Fig. 1D, dotted lines), did not show an enrichment of H2A.Z, ZA, and ZA-rII′. Intergenic regions between two divergent genes, for example, *YCR087C-A* and *ABP1* (Fig. 1D, dashed lines), often contain two separate enrichment peaks of H2A.Z, ZA, and ZA-rII′, supporting the idea that each promoter presents its own enrichment in H2A.Z.

**FIG 2:**
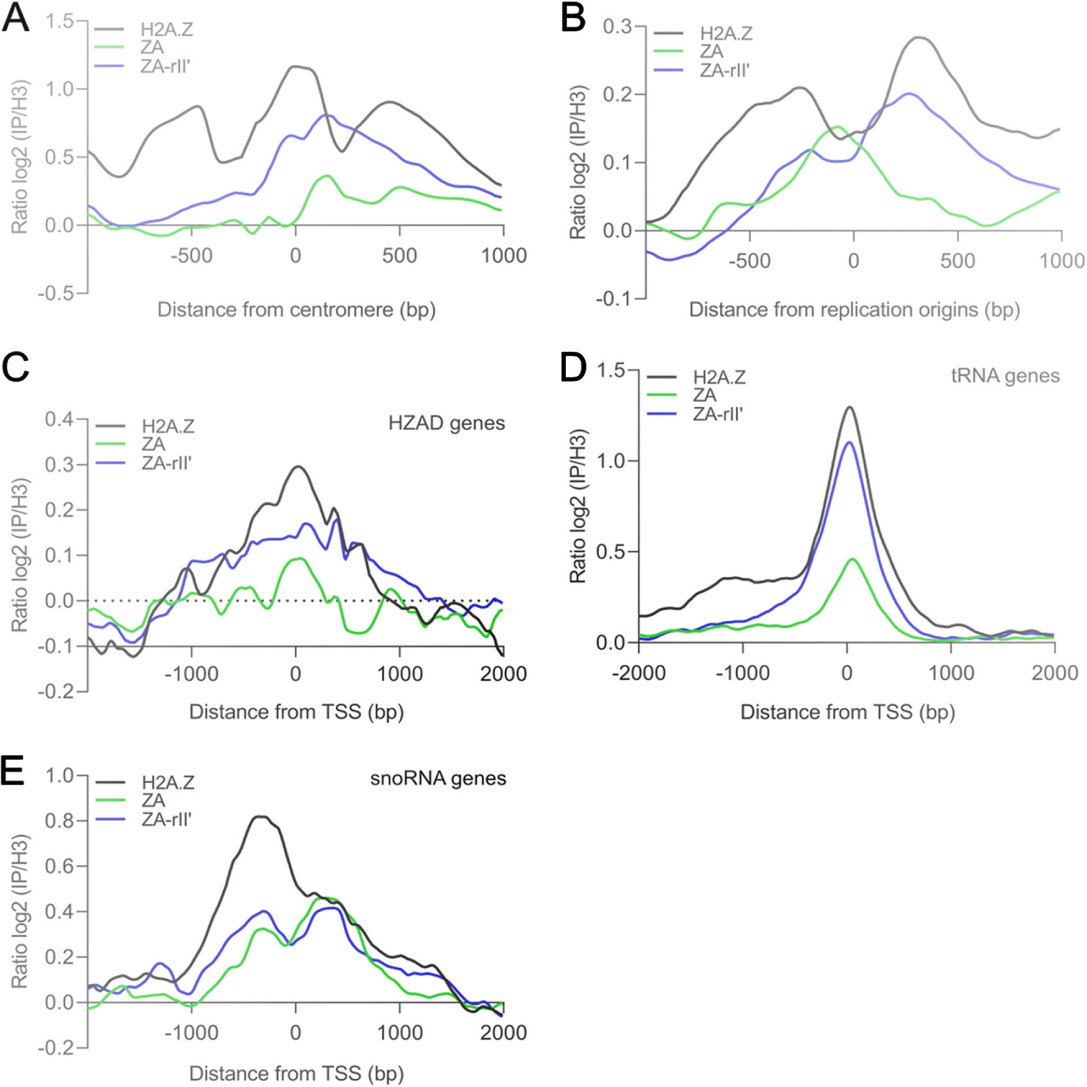
H2A.Z, ZA, and ZA-rII′ occupancies at particular chromosome elements and other transcribed elements. Alignment of genomic data (smoothed H2A.Z occupancy as H2A.Z/H3 log_2_ ratios) with respect to a reference point or coordinates, over particular chromosome elements and other transcribed elements using the Versatile Aggregate Profiler (VAP) tool (40). Representation of the average occupancy of H2A.Z (black line), ZA-rII′ (blue line), and ZA duplicates (green line) over (A) centered centromeres (*n* = 16), (B) centered replication origins (*n* = 352), (C) the transcription start site (TSS) of HZAD (Htz1-activated domain) genes (*n* = 69), (D) the TSS of tRNA genes (*n* = 299), and (E) the TSS of snoRNA genes (*n* = 77) are shown.

H2A.Z is involved in several cellular processes and multiple groups have reported an association of H2A.Z with particular chromosome elements and other transcribed elements (6, 9–12, 14, 29, 30, 32, 35). Given the evidence for these other localizations of H2A.Z, we verified whether its localization at centromeres, replication origins, HZAD (Htz1-activated domain) genes, tRNA genes, and small nucleolar RNA (snoRNA) genes was dependent on its C-terminal region. To do this, we profiled H2A.Z, ZA, and ZA-rII′ localizations (log_2_ ratio IP/H3) over these chromosome elements and the TSS of the respective gene classes using the Versatile Aggregate Profiler (VAP) tool (40). We found that H2A.Z is enriched around centromeres and replication origins, as reported previously, but the H2A.Z distribution pattern at these sites changes significantly when the C-terminal docking domain is modified (Fig. 2A and B), which may affect the protein’s role in chromosome segregation and DNA replication. Moreover, H2A.Z is enriched around the TSS of HZAD, tRNA, and snoRNA genes (Fig. 2C to E), suggesting that the role of H2A.Z in transcription is not limited to Pol II-transcribed genes but also includes Pol III-transcribed genes. Furthermore, replacement of the H2A.Z C-terminal region with the corresponding region from H2A causes a significant loss of the H2A.Z derivative over the HZAD and tRNA gene promoters (Fig. 2C and D). In addition, on the snoRNA promoters, there is a clear and distinct shift in localization for the H2A.Z derivatives (Fig. 2E). Interestingly, the addition of the Gal4 transcriptional activating domain partially restores the localization over the HZAD genes and tRNA genes (Fig. 2C-D), but not the effect on the snoRNA genes (Fig. 2E). We performed paired *t* tests to ensure that the differences in binding efficiency observed among the various derivatives were significant, and in most cases (except for HZAD versus ZA-rII′), *P* values were well below 0.01, suggesting that all differences were significant. The results of these *t* tests are shown in Fig. S2. We conclude that the H2A.Z C-terminal docking domain has a role in the efficient recruitment of H2A.Z not only to promoters of protein-coding genes, but also to promoters of noncoding RNAs and on other chromosomal loci. Furthermore, its localization near centromeres, replication origins, and snoRNA genes is absolutely dependent on the C-terminal docking domain.

### The replacement of the C-terminal region decreases the incorporation level of H2A.Z at target promoters.

As mentioned above, H2A.Z is preferentially localized at promoters and gene regulatory regions, and this localization is conserved from yeasts to mammals (9–21). Although it was originally suggested that H2A.Z is incorporated in the −1 and +1 nucleosomes flanking the nucleosome-free region, more recent data have suggested that H2A.Z is almost exclusively incorporated in the +1 nucleosome in the direction of transcription (16, 23). Moreover, using strains expressing the ZA fusion protein or other variant H2A.Z proteins, previous experiments have documented a lower occupancy in nucleosomes at specific gene promoters, such as those of *PHO5*, *PUR5*, and *GAL* (33–35), suggesting that the incorporation of H2A.Z into nucleosomes at gene promoters is dependent on its C-terminal region.

**FIG 3:**
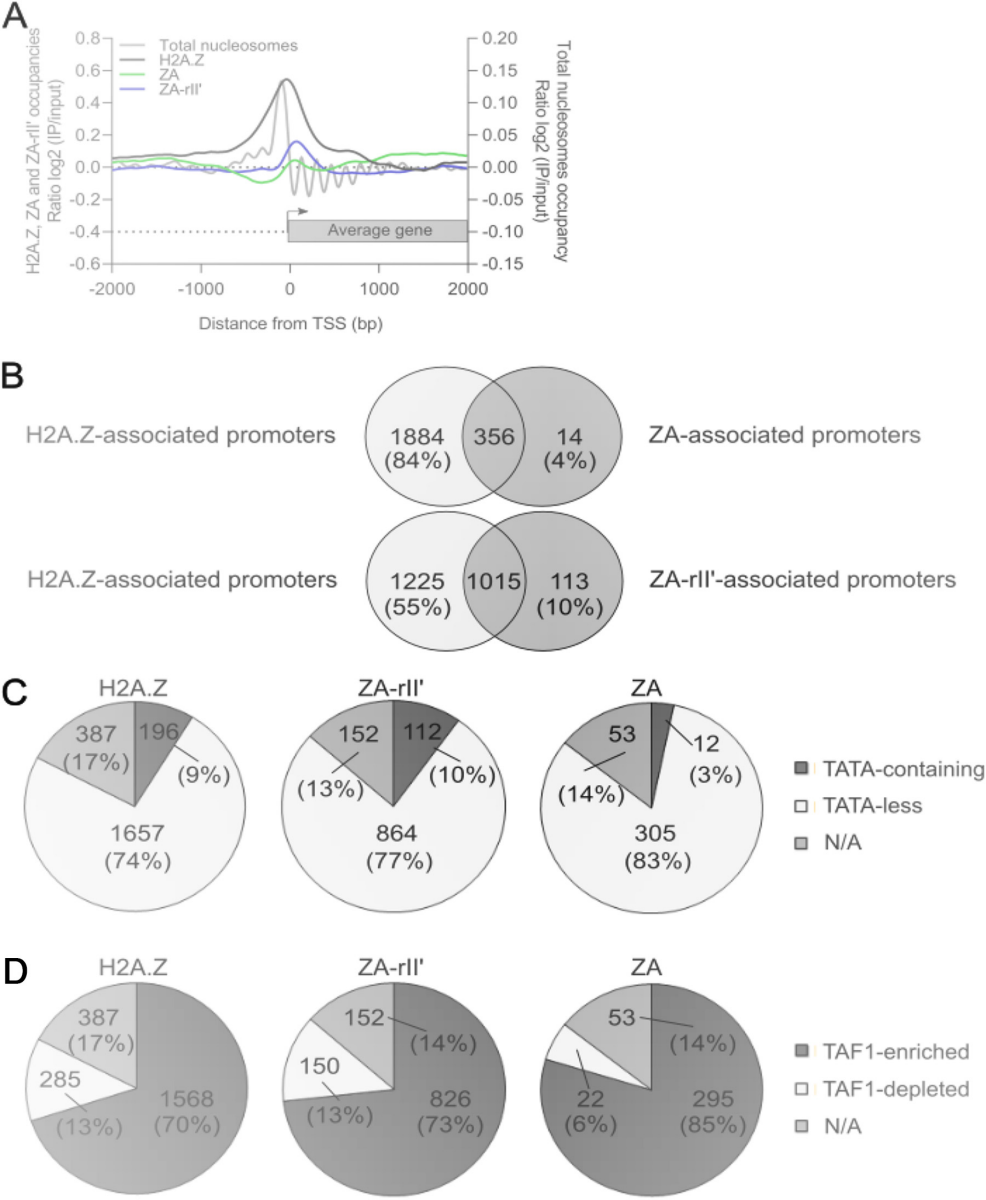
Modification of the C-terminal region decreases the incorporation level of H2A.Z at TAF1-enriched promoters. (A) Alignment of the smoothed data (log_2_ ratios) of H2A.Z, ZA, and ZA-rII′ IPs normalized to input DNA and the total nucleosomes (gray line) over the yeast promoters (data set from Luk et al. [41]). (B) Venn diagram depicting the overlapping and unique H2A.Z-, ZA-, and ZA-rII′-associated genes. The expression of ZA-rII′ partially restored the localization compared to ZA. The vast majority of ZA- and ZA-rII′-associated genes overlap H2A.Z-associated genes. Pie charts showing the number and percentage of H2A.Z-, ZA-rII′-, and ZA-associated gene promoters that are TATA-containing or TATA-less (C); and for gene promoters enriched or depleted for TAF1, a subunit of TFIID (D).

We also measured the occupancy level and mapped the respective proteins over yeast promoters using the VAP tool (log_2_ ratio IP/Input) (40). The enrichment data were aligned for all genes with respect to a reference point, the TSS, and showed that H2A.Z is enriched in a specific area in the promoter regions relative to the coding regions, as expected (Fig. 3A). The detection of ZA-rII′ in this area is significantly reduced with respect to H2A.Z; nevertheless, the addition of the transcriptional activating region to the ZA protein retains the occupation at gene promoters genome-wide at a significant level (Fig. 3A). However, we observed a strongly decreased signal for the ZA protein over these promoters (Fig. 3A), indicating that the occupation level of H2A.Z at promoters is dependent on its C-terminal region. In addition to simple occupancy, we also profiled the positioning of H2A.Z, ZA, and ZA-rII′ nucleosomes and plotted them with respect to the positioning of total nucleosomes (data set from Luk et al. [41]). This allowed us to compare their position with the +1 nucleosome. Given that the +1 nucleosome encompasses the TSS of yeast genes, our data for the WT H2A.Z are in full agreement with previous results, strongly suggestng that H2A.Z enrichment at gene promoters is an incorporation of H2A.Z in the +1 nucleosome. However, the ZA-rII′ peak clearly shifted toward the +2 nucleosome (Fig. 3A, blue versus black curves). In this analysis, due to the extremely low ZA protein incorporation, conclusive determination of the precise sub-localization of the ZA protein was not possible. Taken together, these results support a model in which the H2A.Z C-terminal region is involved in the overall incorporation level of H2A.Z into nucleosomes as well as the precise positioning of the H2A.Z-containing nucleosome on yeast promoters.

Profiling of the H2A.Z, ZA, and ZA-rII′ distributions over promoters using VAP allowed us also to determine the specific number of genes with at least a 2-fold enrichment (IP/H3 log_2_ ratio ≥ 1.00) of H2A.Z, ZA, or ZA-rII′ at the TSS. We identified 2,240 genes that showed an enrichment of H2A.Z at the TSS and referred to them as “H2A.Z-associated genes.” These genes represent 44% of the yeast verified ORFs and are potentially transcriptionally regulated by H2A.Z. We also identified 1,128 ZA-rII′-associated genes (22% of verified ORFs) and 370 ZA-associated genes (7% of verified ORFs). Comparison of the pool of H2A.Z-associated genes with the ZA-associated genes showed that only 4% of ZA-associated genes are specific to this protein and 96% are in the pool of H2A.Z-associated genes (Fig. 3B, upper graph). These data reinforce the conclusion that the replacement of the H2A.Z C-terminal region by the corresponding region from H2A causes a loss of occupancy within chromatin rather than a gross mislocalization of the protein. Similarly, of the 1,128 ZA-rII′-associated genes, only 10% did not overlap the H2A.Z-associated gene pool (Fig. 3B, bottom graph). Therefore, the addition of the Gal4 transcriptional activating domain to the ZA fusion protein partially restores promoter occupancy within chromatin but is not sufficient to complement it to WT H2A.Z levels.

A previous H2A.Z localization study showed that the majority of H2A.Z associated promoters did not contain classical TATA boxes (12). Given that almost all promoter localizations of the protein chimaeras studied here are found in the pool for the WT H2A.Z protein, the data predicted that the fusion proteins preferentially associate with TATA-less promoters. First, our results confirmed that most of the promoters of H2A.Z-associated genes are TATA-less (74%), and most of them also are TAF1 (TFIID)-enriched (70%) (Fig. 3C, left charts). As predicted above, the promoters of ZA-rII′- and ZA-associated genes also are preferentially TATA-less (77% and 83%, respectively) and TFIID-enriched (also 73% and 85%; Fig. 3C, middle and right charts). These results suggest that the main downstream effector on H2A.Z-occupied promoters could be TFIID. However, the fact that the promoters of ZA-associated genes are preferentially TATA-less and TFIID-enriched also suggests that the targeting of H2A.Z to its specific promoters is independent from its C-terminal region.

### The H2A.Z C-terminal domain is important for positive and negative regulation of transcription of genes.

Given that we observed an enrichment of H2A.Z at the promoters of 44% of ORFs and that this incorporation is affected by replacement of the H2A.Z C-terminal region, we hypothesized that the replacement of the H2A.Z C-terminal region deregulates the expression of H2A.Z-associated genes. To verify this hypothesis, we performed RNA sequencing (RNA-seq) to analyze the effect of replacement of the H2A.Z C terminus on gene expression. We compared the transcriptomes of Δ*htz1* cells and Δ*htz1* cells complemented with a copy of H2A.Z, ZA, or ZA-rII′. We identified genes from the Δ*htz1*, ZA, and ZA-rII′ strains showing differential expression with an adjusted *P* value of <0.05 and an absolute log_2_-fold change of >1.00 compared to the WT strain.

**FIG 4:**
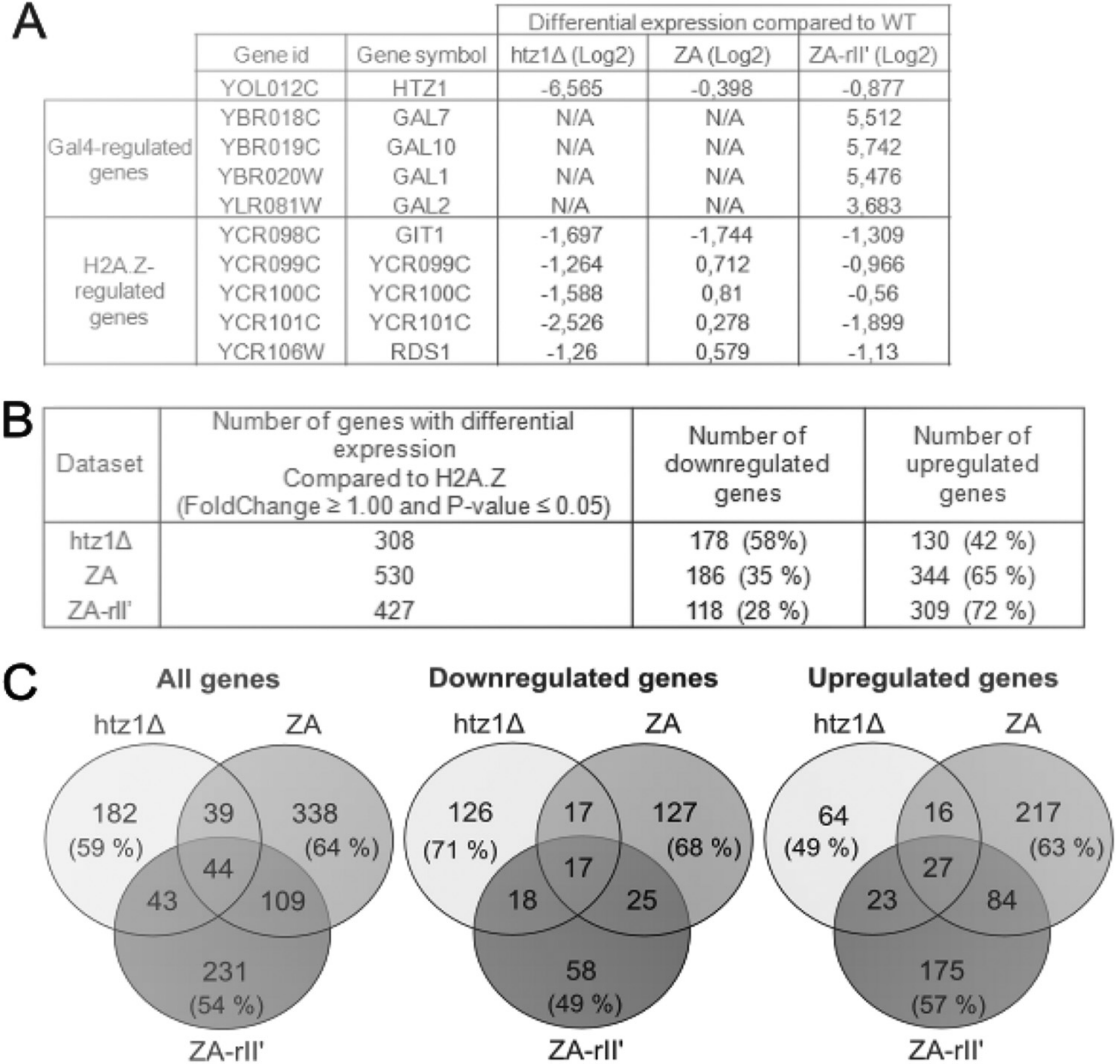
The H2A.Z C terminus is involved in positive and negative regulation of target genes. (A) Expression of known gene sets show the expected changes as assessed by RNA sequencing (RNA-seq). Gal4-regulated genes are listed as controls for expression of genes activated by the Gal4 transcriptional domain (rII′). H2A.Z-regulated genes are also listed as controls. The differential expression (log_2_) of each gene was obtained by comparing the expression in Δ*htz1*, ZA, or ZA-rII′ strains to the Δ*htz1* strain complemented with a copy of *HTZ1*. (B) Number of differentially expressed genes (log_2_-fold change ≥ 1.00, *P* ≤ 0.05) for Δ*htz1*, ZA, and ZA-rII′ strains compared to the wild-type (WT) strain are listed, as well as the number and percentage of downregulated and upregulated gene for each strain. (C) Venn diagrams showing the number of overlapping differentially expressed genes (log_2_-fold change ≥ 1.00) among Δ*htz1*, ZA, and ZA-rII′ strains compared to the WT. Diagrams show overlaps among all genes, downregulated genes, and upregulated genes. Percentages of unique genes for a strain are relative to the total number of genes, the total number of downregulated or upregulated genes for that strain.

As a control, we wanted to verify whether RNA expression levels would recapitulate previous results which showed that differential expression of Gal4-regulated genes is higher in cells harboring the ZA-rII′ construct compared to WT cells, and that the expression of known H2A.Z-regulated genes is downregulated in Δ*htz1* cells (6, 35) (Fig. 4A). Using RNA-seq, 308 differentially expressed genes (log_2_-fold change ≥ 1.00 and *P* ≤ 0.05) were identified in the Δ*htz1* strain compared to the WT, 530 differentially expressed genes in the ZA strain, and 427 differentially expressed genes in the ZA-rII′ strain (Fig. 4B). While a slight bias toward downregulated genes (58%) was observed in the Δ*htz1* strain, in the ZA- and ZA-rII′-expressing strains, 65% and 72%, respectively, of the differentially expressed genes were upregulated. This upregulation of genes in the ZA-rII′ strain would be expected because we inserted a transcriptional activating domain into the ZA fusion protein. Remarkably, however, a direct comparison of the identity of the differentially expressed genes in the Δ*htz1*, ZA, and ZA-rII′ strains allowed to determine that half or more of the differentially expressed genes were specific to each strain (Fig. 4C, left). This surprising pattern held true for downregulated and upregulated genes (Fig. 4C, middle and right). Therefore, these analyses show that the changes in the transcriptomes in the respective cells diverged considerably. Therefore, replacement of the H2A.Z C terminus causes a major shift of the expressed transcriptome compared to a WT or Δ*htz1* strain.

Given that H2A.Z is associated with several biological processesm such as transcription, DNA repair, chromosome segregation, chromatin silencingm and RNA splicing (7), we used the Metascape tool to identify significant enrichment of Gene Ontology terms among differentially expressed genes in Δ*htz1*, ZA, and ZA-rII′ strains compared to the WT. Although we found some enriched biological processes for differentially expressed genes in the ZA and ZA-rII′ strains compared to the WT, we detected no significant distinction between gene classes which would allow us to draw any convincing conclusion regarding the role of the H2A.Z C-terminal region.

### H2A.Z and H2A.Z derivatives incorporation at the +1 nucleosome of genes does not correlate with differential gene expression.

**FIG 5:**
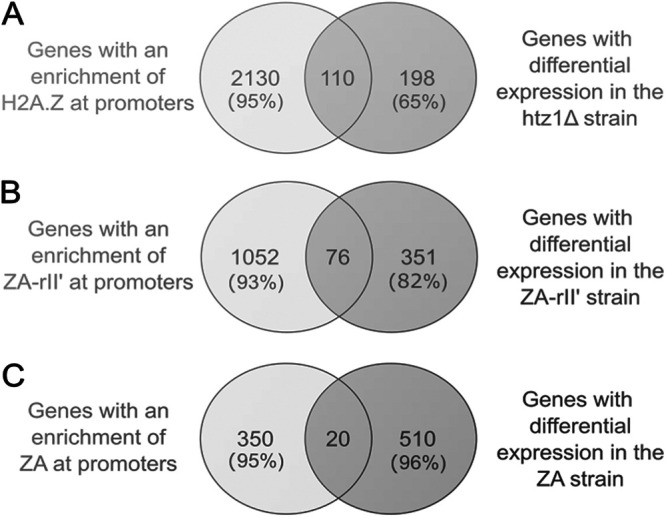
Expression levels of genes with an enrichment of H2A.Z or H2A.Z derivatives on their promoters does not change. Venn diagrams show the number of overlapping genes between the occupancy at promoters and differentially expressed genes. (A) Diagram depicting the overlap between genes with an enrichment of H2A.Z at promoters and differentially expressed genes in the Δ*htz1* strain compared to the WT strain. (B) Same as panel A, but for the ZA-rII′ construct. (C) Same as panel A, but for the ZA construct. Percentages show the number of genes that do not overlap relative to the total number of occupied genes of differentially expressed genes.

Previous studies have reported a negative correlation between H2A.Z occupancy at promoters and transcription rates in yeast (4, 9, 11, 12). Because of this observation, it has been suggested that H2A.Z marks the promoters of inactive or weakly transcribed genes and presumably prepares nucleosomes for disassembly upon gene induction (4, 5, 9, 26). However, the level of acetylated H2A.Z at promoters has been associated with actively transcribed genes (13). In contrast, several papers have reported that the H2A.Z incorporation level at gene promoters does not correlate with their transcription rates or RNA polymerase II occupancy (10, 16, 29). To investigate whether there was a correlation between the H2A.Z occupancy at the +1 nucleosome and differential gene expression in our data, we compared the data sets of genes with an enrichment of H2A.Z or H2A.Z derivatives at the promoter (ChIP-chip data) and the differentially expressed genes (RNA-seq data) (Fig. 5). The results from this comparison suggest that in the Δ*htz1* strain, 65% of differentially expressed genes did not show an enrichment of H2A.Z at their promoter (Fig. 5A). Similarly, 82% and 96% of differentially expressed genes in the ZA-rII′- and ZA-expressing strains did not show an enrichment of ZA-rII′ or ZA at their promoter (Fig. 5B–C). These results suggest that the association of the proteins studied here at specific promoters is inversely correlated with the expression level of the gene.

**FIG 6:**
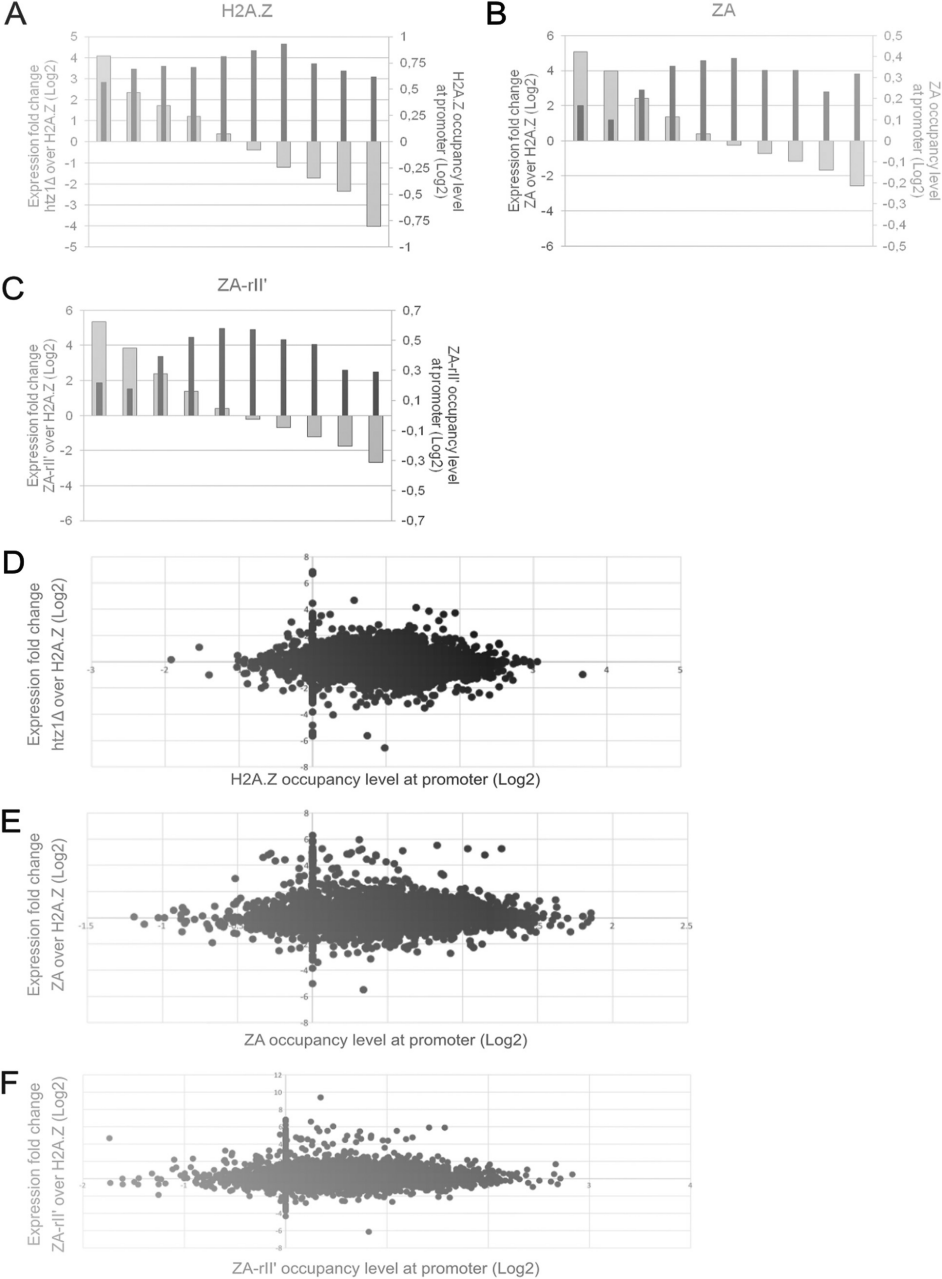
H2A.Z and H2A.Z derivatives incorporation at the +1 nucleosome of genes does not correlate with differential gene expression. (A to C) All genes were sorted by differential expression level (log_2_-fold change over H2A.Z strain) (gray bars), separated into 10 groups of decreasing expression level, and compared to the average protein occupancies at the TSS for the same gene group (log_2_ ratio) (black bars). (D and F) H2A.Z and H2A.Z derivative occupancies at the TSS of all genes (log_2_ ratio) plotted against differential gene expression values (log_2_-fold change over H2A.Z strain).

To examine this hypothesis more directly, we ranked the genes into 10 groups based on their differential gene expression level and compared them to H2A.Z or H2A.Z derivative occupancy at the respective promoter (Fig. 6A to C). Intriguingly, we found no correlation between the incorporation level of H2A.Z and H2A.Z derivatives at promoters and differential gene expression levels, possibly suggesting that genes with very high differential expression (fold change over the WT strain) showed low levels of H2A.Z or H2A.Z derivative occupancy at promoters, and vice versa. However, when the H2A.Z and H2A.Z derivatives’ occupancy at the TSS of all genes (log_2_ ratio) was plotted against ranked differential gene expression values (log_2_-fold change over H2A.Z strain) (Fig. 6D and F), no correlation between the occupancy at promoters and the expression level of the gene was observed, confirming previous studies (10, 16, 29). Thus, we propose that H2A.Z can be targeted and recruited to promoters of transcriptionally active and inactive genes, independently from its C-terminal region, but the transcriptional outcome of a specific gene will differ in a H2A.Z C-terminal-modified strain due to as-yet unknown mechanisms.

### ZA and ZA-RII′ require SWR1-C for deposition within chromatin loci.

**FIG 7:**
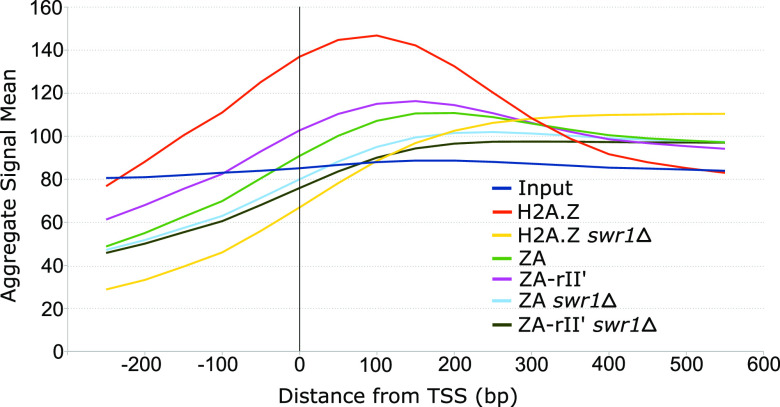
ZA and ZA-rII′ require SWR1-C for deposition within chromatin loci. Chromatin immunoprecipitation sequencing (ChiP-seq) of H2A.Z, ZA, and ZA-rII′ in either WT or Δ*swr1* cells. ChIP-seq reads were analyzed using the GenPipes chipseq pipeline (59). Figure shows enrichment of all H2A.Z derivatives relative to the TSS.

One important issue that needed to be addressed was whether or not the differential association of H2A.Z derivatives to chromatin would mean that they would no longer depend on physical association to SWR1-C, an important H2A.Z chaperone, and would therefore function through a different chromatin-depositing factor. To address this, we performed ChIP-seq experiments using HA-tagged versions of H2A.Z, ZA, and ZA-RII′ in wild-type cells or cells lacking SWR1 (Δ*swr1*). We reasoned that if ZA, particularly ZA-rII′, functioned independently of SWR1-C, their association to gene regulatory regions would not be significantly affected by the loss of SWR1-C. Figure 7 shows the average enrichment of H2A.Z derivatives relative to the TSS of genes in either WT or Δ*swr1* cells. As expected, WT H2A.Z shows the strongest association to the TSS region of genes, followed by ZA-RII′ and then ZA. Importantly, and in all three cases, there is a loss of significant association of the H2A.Z derivatives to the TSS region of genes in the absence of the SWR1 complex. These results clearly demonstrate that differential association of H2A.Z derivatives to chromatin regions cannot be attributed to a problem with their association to the main H2A.Z chaperone complex.

## DISCUSSION

The aim of this study was to determine whether the C terminus of H2A.Z affects the nucleosomal occupancy level of this alternative histone and/or the precise localization of these nucleosomes. While some directed analyses on a few loci have been reported, here, we analyzed these effects genome-wide and hence for all possible H2A.Z nucleosomes. For this purpose, we used two H2A.Z variants lacking the normal C terminus: one which the C terminus was replaced with that of H2A (ZA protein), and one which additionally contained an acidic activation domain (ZA-rII′ protein). We performed ChIP-chip on these two variant proteins and compared the results of the chimaera proteins with those of WT H2A.Z. First of all, the overall localization of the WT H2A.Z reassessed here coincides almost perfectly with previous data (9–12, 14, 29). Therefore, we are confident that the differential results with the chimaera adequately covered all concerned loci. Second, the incorporation efficiency globally appeared to be much reduced for the ZA protein, with a slight amelioration for the ZA-rII′ protein (Fig. 1). However, upon closer inspection, there are major differences in these results depending on the locus analyzed.

### RNA Pol II promoters: expected and unexpected effects.

As expected (33), ZA protein occupancy in the +1 nucleosome after the nucleosome-free region is virtually undetectable, compared to H2A.Z. The rII’ addition complements this loss somewhat, but not back to WT levels. Interestingly, overall, the replacement of the H2A.Z C-terminal region did not change the targeting on specific promoters: virtually all affected loci are a subset of the loci with H2A.Z. There was no correlation between the differential expression level of a gene and the occupancy level at the TSS (Fig. 4), as expected from previous studies (10, 16, 29). Given that steady-state RNA levels reflect a combination of transcription initiation rate and RNA turnover rates (16), we suggest that the occupancy level of H2A.Z at promoters does not predict the expression level of a gene and that H2A.Z marks the promoters of active and inactive genes. Multiple explanations could be provided on how the replacement of the H2A.Z C-terminal domain might affect the transcriptional outcome of a gene, for example, impaired recruitment of certain transcriptional mediators or components of the transcriptional machinery, such as TFIID, SAGA, and RNA Pol II (5, 8). However, the +1 nucleosome enrichment peak of ZA-rII′ shifted significantly toward the +2 nucleosome position; due to its very low occupancy, it is not clear whether the ZA +1 nucleosome shifted (Fig. 3B). How the ZA-rII′ shift would affect local or global gene transcription remains to be determined. It is also possible that ZA and ZA-rII′ could affect chromatin dynamics and gene regulation via an indirect mechanism, i.e., independent from that elicited at the TSS of a gene.

### RNA Pol III promoters and HZAD loci.

The changes in localization patterns of H2A.Z derivatives over RNA Pol III-transcribed tRNA genes are remarkably similar to Pol II promoters; that is, the replacement of the H2A.Z C-terminal region caused a reduced occupancy of H2A.Z derivatives at the +1 nucleosome at tRNA promoters and the rII′ addition complements this loss almost back to WT levels.

HZAD loci are large areas encompassing at least several nucleosomes that are enriched in H2A.Z. Again, the ZA derivative is almost completely lost from these areas and the rII′ peptide does rescue this loss somewhat (Fig. 2C); thus, they behave very similar to RNA Pol II and RNA Pol III promoters.

### snoRNA gene promoters.

While snoRNA genes are transcribed by RNA polymerase II, there was clearly a different effect upon the replacement of the H2A.Z C-terminal region. First, the reduction in occupancy at the +1 nucleosome was similar for both H2A.Z derivatives, thus with no recovery for the ZA-rII′ protein. Second, the ZA- and ZA-rII′-nucleosomes appeared in two distinct peaks on either side of the TSS compared to one strong enrichment peak upstream of the TSS for the WT H2A.Z (Fig. 2E). At present, we do not have a good explanation for this surprisingly differential effect on snoRNA gene promoters.

### Centromeres and origins of replication.

There was clearly a different distribution of the H2A.Z derivatives in the chromatin surrounding centromeres and replication origins compared to WT H2A.Z (Fig. 2A–B). For example, while H2A.Z is incorporated in the centromeric and pericentromeric nucleosomes, ZA- and ZA-rII′ are incorporated only on one side of the centromere. Moreover, the double peak of H2A.Z nucleosomes surrounding replication origins is reduced to one peak of ZA nucleosomes right at the center of the origin. This is interesting because yeast cells expressing the ZA fusion display a lower growth rate than WT H2A.Z and ZA-rII′ (33). Thus, the fact that ZA associates with the center of replicating origins could impede or slow down the recruitment of origin factors.

### How could such disparate, locus-specific effects be explained?

Previous papers have shown that the H2A.Z C-terminal region determines the association of H2A.Z with the Chz1 histone chaperone or the SWR1 complex for its deposition into chromatin (34, 38, 42, 43). Therefore, the generally reduced occupancy signal of H2A.Z derivatives could be due to an impaired binding of H2A.Z derivatives to SWR-C or a problem in the deposition of H2A.Z derivatives. However, we were able to show that in yeast cells bearing a deletion in *SWR1* (Δ*swr1*), the binding of all H2A.Z chimeras was nearly abolished, suggesting that ZA and ZA-RII′ work primarily through SWR1-C for their genomic localization. This is also in line with a recent study by Brewis et al. (44) who used H2A-H2A.Z chimeras to demonstrate that the M6 region of H2A.Z was necessary and sufficient for interaction with SWR1-C. They also showed that the H2A.Z C-terminal region (including the M6 region) was not sufficient to rescue H2A.Z occupancy at specific gene promoters. This is consistent with the fact that we observed differences in localization with our ZA and ZA-rII′ derivatives. Moreover, anchor-away experiments showed that an impairment of the SWR-C mediated deposition of H2A.Z still results in a certain level of H2A.Z occupancy within chromatin (25), possibly explaining the observed occupancy of ZA and ZA-rII′ genome-wide.

Previous papers have also demonstrated that yeast and mammalian nucleosomes containing H2A.Z with mutations in their C terminus are less stable, i.e., the C terminus is required for stable retention of H2A.Z (22, 36, 37, 45). Thus, ZA- or ZA-rII′-containing nucleosomes may be less stable and more easily lost from chromatin.

In addition to problems in deposition and nucleosome stability, the eviction mechanisms for H2A.Z derivatives from chromatin might vary depending on localization. While there is evidence that the INO80 remodeling complex contributes to H2A.Z eviction in general (37, 46), its eviction at gene promoters is linked to transcription initiation, promoter escape, and early elongation of Pol II (25, 47).

There is also evidence that H2A.Z is deposited via replication-dependent random incorporation into the genome and selectively depleted from transcription units (26–28, 48). This hypothesis could provide a valid explanation for the presence or accumulation of H2A.Z in regions such as HZAD genes, origins of replication, and centromeres. In that case, the surrounding chromatin could influence the final localization of variant-containing nucleosomes, depending on chromatin remodelers attracted to the area via other histone modifications. These remodelers have significant conserved roles in nucleosome homeostasis (49). For example, at Pol II gene promoters, the position of the +1 nucleosome is set by the remodeling activities of Isw2, Isw1a, Ino80, and general gene regulatory factors (45, 50–52). Because HZADs and snoRNA genes are mostly Pol II-transcribed genes as well, we suggest that the precise localization of the H2A.Z-containing +1 nucleosome in those areas could be dependent on a similar set of factors. Isw1, Isw2, and Ino80 have also been reported to be localized to tRNA gene promoters (53, 54), and INO80-C is present at various chromosome elements, including origins of replication and pericentric chromatin (54–56). Furthermore, specific histone tail modifications are known to attract other remodeling complexes, such as the SWI/SNF and the CHD families (49). While the localization of these complexes on the yeast genome is consistent with a role in regulating nucleosomes with H2A.Z, locus-specific regulation mechanisms remain unclear.

The C-terminal-modified H2A.Z variants used in this study do not, to our knowledge, represent physiological proteins in yeast. Nevertheless, we note that there is a naturally occurring splice isoform of the human H2A.Z-2 gene encoding a C-terminally truncated H2A.Z protein which is predominantly expressed in human brain, skeletal muscle, and liver tissues (36). This short form of human H2A.Z-2 protein is less stably bound to chromatin than the full-length protein (36), suggesting that the function of the H2A.Z C-terminal region in the regulation of the association of H2A.Z with nucleosomes is conserved and biologically relevant.

Overall, our study revealed that there is no global, all-encompassing mechanism by which the H2A.Z C terminus affects nucleosomal properties. It appears that incorporation, localization, and H2A.Z loss rates vary in a context-specific fashion. Therefore, the locus-specific mechanisms by which the C terminus of H2A.Z affects incorporation rates and localization need to be investigated by targeted and locus-specific approaches.

## MATERIALS AND METHODS

### Yeast strains and protein chimaera construction.

The WT haploid strain W303 (MAT A) was used in this study. The Δ*htz1* haploid strains was obtained by replacing the *HTZ1* allele with a KMX cassette. The HA-H2A.Z, HA-ZA, and HA-ZA-rII′ constructs used in this study are as described previously (5, 33). Briefly, all H2A.Z derivatives bear a HA tag inserted in the BglII site of the *HTZ1* gene and were expressed from the *ACT1* promoter. The ZA fusion was constructed by fusing amino acids 91 to 132 of H2A to the C-terminal region of amino acids 1 to 97 of H2A.Z (5). The ZA-rII′ fusion was generated by adding amino acids 840 to 881 of Gal4 to the C-terminal end of ZA (33). The plasmids were linearized and integrated at the endogenous *URA3* locus of the Δ*htz1* strain.

### Chromatin immunoprecipitations, ChIP-chip, and ChIP-seq.

ChIP experiments were performed in duplicates as previously described (5, 9). Briefly, 50 mL of cells were grown in yeast extract-peptone-dextrose (YPD; 2% glucose) medium to an optical density at 600 nm (OD_600_) of 0.6 and fixed with 1% formaldehyde for 10 min. Next, 350 μL of sonicated whole-cell extract was incubated with either anti-HA (12CA5; Roche) or anti-H3 (ab1791; Abcam) antibody coupled to magnetic beads (Dynal) overnight at 4°C with agitation. Immunoprecipitated DNA was used for genome-wide location analysis.

For the ChIP-chip, H2A.Z, ZA, and ZA-rII′ ChIP DNA, labeled with Cy5, was hybridized in competition with Cy3-labeled H3 ChIP or input DNA. The microarrays used for location analysis have been described previously (57) and were purchased from Agilent Technologies (Palo Alto, CA, USA). Samples were co-hybridized on an array containing a total of 180,000 60-mer probes (including controls) covering the entire yeast genome with virtually no gaps between the probes.

For the ChIP-seq, ChIP experiments were performed in duplicates as previously described (57). For anti-HA ChIP-seq, 500 μL of whole-cell extract was incubated with 50 μL pre-equilibrated Pierce Anti-HA Magnetic Beads (Thermo Fisher Scientific [Waltham, MA], cat no. 88836) overnight at 4°C. Immunoprecipitated DNAs were purified and used as input into standard Illumina library construction.

### ChIP-chip data analysis.

The ChIP-chip data were normalized using the Limma Loess method, and replicates were combined using a weighted average method as described previously (58). The data were subjected to one round of smoothing using a Gaussian filter (SD = 100 bp). Aggregate profiles were generated using the VAP (40). All data sets for the ChIP-chip experiment were deposited into the NCBI Gene Expression Omnibus (GEO) database under accession no. GSE156492 (enter token gzozeeimrrqlrqd into the box).

### ChIP-seq data analysis.

ChIP-seq reads were analyzed using the GenPipes ChIP-seq pipeline (59). Briefly, the pipeline begins by trimming adaptors and low-quality bases using Trimmomatic v0.39 (60) and mapping the reads to a Saccharomyces cerevisiae R64-1-1 reference genome using Burrows-Wheeler Aligner (BWA) v0.7.17 (61). Reads are filtered by mapping quality and duplicate reads are marked using Sambamba v0.8.0 (62). Next, Homer v4.11 quality control routines were used to provide information and feedback regarding the quality of the experiment (63). Peak calls are executed by Model-Based Analysis of ChIP-seq (MACS) v2.2.7.1 (64), and annotation and motif discovery for narrow peaks are executed using Homer.

- Reads were analyzed using the GenPipes ChIP-seq pipeline (https://bitbucket.org/mugqic/genpipes/src/master/pipelines/chipseq/) as follows:
  - Trimmomatic v0.39 adapters.fa:2:30:15 trailing:30 minlen:50
  - BWA -k 100000000 -v 3 -t 7 -Y Saccharomyces_cerevisiae.R64-1-1.fa
  - Sambamba markdup & view to mark duplicate reads and filter to keep reads with mapping quality > 20
  - Homer suites (makeTagDirectory, makeUCSCfile)
  - Macs2 callpeak -fix-bimodal -gsize 9725684.0 -nolambda
  - Homer annotatePeaks.pl -gsize 9725684.0 -cons -CpG
  - Homer findMotifsGenome.pl
- Download VAP from https://bitbucket.org/labjacquespe/vap_interface/downloads/vap-2.0.0-alpha3.dmg
- Annotation GTF from Saccharomyces_cerevisiae R64 downloaded from Ensembl release 77 (http://ftp.ensembl.org/pub/release-77/gtf/saccharomyces_cerevisiae/Saccharomyces_cerevisiae.R64-1-1.77.gtf.gz)
- Annotation filtered for gene_biotype = “protein_coding”
- Filtered annotation converted to genepred using ucsc utilities v346
- VAP was used to analyse sample bigwig files to generate aggregates profiles using using default values except the following configuration:
  - Filtered annotation for coding genes
  - # windows/block: 10(ups)-10(inter)-25(ref)-6(inter)-6(down)
  - Windows to smooth: 6
  - # dataset/graph: all

### RNA-seq and differential expression analysis.

The RNA-seq analysis was performed in triplicates on a Δ*htz1* strain and on Δ*htz1* strains with an integrated copy of the *HTZ1* gene or the ZA or ZA-rII′ constructions. Total RNA extraction was extracted using the Direct-zol RNA MiniPrep kit (Zymo Research) according to the manufacturer’s specifications. Illumina sequencing libraries were prepared using 3′-B and 5′-A adaptors, as well as IGA-PCR-PE-F, TruSeq-MPEX-R, DSN-TruSeq-F, and DSNTruSeq-R primers. The trimmed reads were aligned using Tophat37 onto a genome composed of the S. cerevisiae S288C genome (sacCer3). DESeq2 was used to identify the differentially expressed genes. Yeast genes showing a differential expression with an adjusted *P* value of <0.05 and having an absolute log_2_-fold change of >1.00 were considered differentially expressed. The Gene Ontology term enrichment analysis for differentially expressed genes was investigated using Metascape (https://metascape.org/gp/index.html#/main/step1). All data sets for the RNA-seq experiment were deposited into NCBI GEO database under accession no. PRJNA657613.

### Data availability.

The raw reads and processed files from the ChIP-chip, ChIP-seq, and RNA-seq experiments have been deposited in the NCBI Gene Expression Omnibus (GEO) database (http://www.ncbi.nlm.nih.gov/geo/) and are accessible under accession no. GSE156492 and PRJNA657613.

## References

[1] Talbert PB, Henikoff S. 2017. Histone variants on the move: substrates for chromatin dynamics. Nat Rev Mol Cell Biol 18:115–126. doi:10.1038/nrm.2016.148. 27924075

[2] Leach TJ, Mazzeo M, Chotkowski HL, Madigan JP, Wotring MG, Glaser RL. 2000. Histone H2A.Z is widely but nonrandomly distributed in chromosomes of *Drosophila melanogaster*. J Biol Chem 275:23267–23272. doi:10.1074/jbc.M910206199. 10801889

[3] Dhillon N, Kamakaka RT. 2000. A histone variant, Htz1p, and a Sir1p-like protein, Esc2p, mediate silencing at HMR. Mol Cell 6:769–780. doi:10.1016/S1097-2765(00)00076-9. 11090616

[4] Santisteban MS, Kalashnikova T, Smith MM. 2000. Histone H2A.Z regulates transcription and is partially redundant with nucleosome remodeling complexes. Cell 103:411–422. doi:10.1016/s0092-8674(00)00133-1. 11081628

[5] Adam M, Robert F, Larochelle M, Gaudreau L. 2001. H2A.Z Is Required for global chromatin integrity and for recruitment of RNA polymerase II under specific conditions. Mol Cell Biol 21:6270–6279. doi:10.1128/MCB.21.18.6270-6279.2001. 11509669 PMC87352

[6] Meneghini MD, Wu M, Madhani HD. 2003. Conserved histone variant H2A.Z protects euchromatin from the ectopic spread of silent heterochromatin. Cell 112:725–736. doi:10.1016/s0092-8674(03)00123-5. 12628191

[7] Zlatanova J, Thakar A. 2008. H2A.Z: view from the top. Structure 16:166–179. doi:10.1016/j.str.2007.12.008. 18275809

[8] Suto RK, Clarkson MJ, Tremethick DJ, Luger K. 2000. Crystal structure of a nucleosome core particle containing the variant histone H2A.Z. Nat Struct Biol 7:1121–1124. doi:10.1038/81971. 11101893

[9] Guillemette B, Bataille AR, Gévry N, Adam M, Blanchette M, Robert F, Gaudreau L. 2005. Variant histone H2A.Z is globally localized to the promoters of inactive yeast genes and regulates nucleosome positioning. PLoS Biol 3:e384. doi:10.1371/journal.pbio.0030384. 16248679 PMC1275524

[10] Raisner RM, Hartley PD, Meneghini MD, Bao MZ, Liu CL, Schreiber SL, Rando OJ, Madhani HD. 2005. Histone variant H2A.Z marks the 5′ ends of both active and inactive genes in euchromatin. Cell 123:233–248. doi:10.1016/j.cell.2005.10.002. 16239142 PMC2039754

[11] Li B, Pattenden SG, Lee D, Gutiérrez J, Chen J, Seidel C, Gerton J, Workman JL. 2005. Preferential occupancy of histone variant H2AZ at inactive promoters influences local histone modifications and chromatin remodeling. Proc Natl Acad Sci USA 102:18385–18390. doi:10.1073/pnas.0507975102. 16344463 PMC1317944

[12] Zhang H, Roberts DN, Cairns BR. 2005. Genome-wide dynamics of Htz1, a histone H2A variant that poises repressed/basal promoters for activation through histone loss. Cell 123:219–231. doi:10.1016/j.cell.2005.08.036. 16239141 PMC2788555

[13] Millar CB, Xu F, Zhang K, Grunstein M. 2006. Acetylation of H2AZ Lys 14 is associated with genome-wide gene activity in yeast. Genes Dev 20:711–722. doi:10.1101/gad.1395506. 16543223 PMC1413291

[14] Albert I, Mavrich TN, Tomsho LP, Qi J, Zanton SJ, Schuster SC, Pugh BF. 2007. Translational and rotational settings of H2A.Z nucleosomes across the *Saccharomyces cerevisiae* genome. Nature 446:572–576. doi:10.1038/nature05632. 17392789

[15] Chereji RV, Ramachandran S, Bryson TD, Henikoff S. 2018. Precise genome-wide mapping of single nucleosomes and linkers *in vivo*. Genome Biol 19:19. doi:10.1186/s13059-018-1398-0. 29426353 PMC5807854

[16] Bagchi DN, Battenhouse AM, Park D, Iyer VR. 2020. The histone variant H2A.Z in yeast is almost exclusively incorporated into the +1 nucleosome in the direction of transcription. Nucleic Acids Res 48:157–170. doi:10.1093/nar/gkz1075. 31722407 PMC7145542

[17] Barski A, Cuddapah S, Cui K, Roh T-Y, Schones DE, Wang Z, Wei G, Chepelev I, Zhao K. 2007. High-resolution profiling of histone methylations in the human genome. Cell 129:823–837. doi:10.1016/j.cell.2007.05.009. 17512414

[18] Mavrich TN, Jiang C, Ioshikhes IP, Li X, Venters BJ, Zanton SJ, Tomsho LP, Qi J, Glaser RL, Schuster SC, Gilmour DS, Albert I, Pugh BF. 2008. Nucleosome organization in the *Drosophila* genome. Nature 453:358–362. doi:10.1038/nature06929. 18408708 PMC2735122

[19] Bruce K, Myers FA, Mantouvalou E, Lefevre P, Greaves I, Bonifer C, Tremethick DJ, Thorne AW, Crane-Robinson C. 2005. The replacement histone H2A.Z in a hyperacetylated form is a feature of active genes in the chicken. Nucleic Acids Res 33:5633–5639. doi:10.1093/nar/gki874. 16204459 PMC1243646

[20] Zilberman D, Coleman-Derr D, Ballinger T, Henikoff S. 2008. Histone H2A.Z and DNA methylation are mutually antagonistic chromatin marks. Nature 456:125–129. doi:10.1038/nature07324. 18815594 PMC2877514

[21] Gévry N, Chan HM, Laflamme L, Livingston DM, Gaudreau L. 2007. p21 Transcription is regulated by differential localization of histone H2A.Z. Genes Dev 21:1869–1881. doi:10.1101/gad.1545707. 17671089 PMC1935026

[22] Subramanian V, Mazumder A, Surface LE, Butty VL, Fields PA, Alwan A, Torrey L, Thai KK, Levine SS, Bathe M, Boyer LA. 2013. H2A.Z acidic patch couples chromatin dynamics to regulation of gene expression programs during ESC differentiation. PLoS Genet 9:e1003725. doi:10.1371/journal.pgen.1003725. 23990805 PMC3749939

[23] Rhee HS, Bataille AR, Zhang L, Pugh BF. 2014. Subnucleosomal structures and nucleosome asymmetry across a genome. Cell 159:1377–1388. doi:10.1016/j.cell.2014.10.054. 25480300 PMC4258235

[24] Tramantano M, Sun L, Au C, Labuz D, Liu Z, Chou M, Shen C, Luk E. 2016. Constitutive turnover of histone H2A.Z at yeast promoters requires the preinitiation complex. Elife 5. doi:10.7554/eLife.14243. PMC4995100 27438412

[25] Ranjan A, Nguyen VQ, Liu S, Wisniewski J, Kim JM, Tang X, Mizuguchi G, Elalaoui E, Nickels TJ, Jou V, English BP, Zheng Q, Luk E, Lavis LD, Lionnet T, Wu C. 2020. Live-cell single particle imaging reveals the role of RNA polymerase II in histone H2A.Z eviction. Elife 9. doi:10.7554/eLife.55667. PMC7259955 32338606

[26] Hardy S, Jacques P-E, Gévry N, Forest A, Fortin M-E, Laflamme L, Gaudreau L, Robert F. 2009. The euchromatic and heterochromatic landscapes are shaped by antagonizing effects of transcription on H2A.Z deposition. PLoS Genet 5:e1000687. doi:10.1371/journal.pgen.1000687. 19834540 PMC2754525

[27] Jeronimo C, Watanabe S, Kaplan CD, Peterson CL, Robert F. 2015. The histone chaperones FACT and Spt6 restrict H2A.Z from intragenic locations. Mol Cell 58:1113–1123. doi:10.1016/j.molcel.2015.03.030. 25959393 PMC4475440

[28] Jeronimo C, Robert F. 2016. Histone chaperones FACT and Spt6 prevent histone variants from turning into histone deviants. Bioessays 38:420–426. doi:10.1002/bies.201500122. 26990181

[29] Krogan NJ, Keogh M-C, Datta N, Sawa C, Ryan OW, Ding H, Haw RA, Pootoolal J, Tong A, Canadien V, Richards DP, Wu X, Emili A, Hughes TR, Buratowski S, Greenblatt JF. 2003. A Snf2 family ATPase complex required for recruitment of the histone H2A variant Htz1. Mol Cell 12:1565–1576. doi:10.1016/s1097-2765(03)00497-0. 14690608

[30] Krogan NJ, Baetz K, Keogh M-C, Datta N, Sawa C, Kwok TCY, Thompson NJ, Davey MG, Pootoolal J, Hughes TR, Emili A, Buratowski S, Hieter P, Greenblatt JF. 2004. Regulation of chromosome stability by the histone H2A variant Htz1, the Swr1 chromatin remodeling complex, and the histone acetyltransferase NuA4. Proc Natl Acad Sci USA 101:13513–13518. doi:10.1073/pnas.0405753101. 15353583 PMC518788

[31] Keogh M-C, Mennella TA, Sawa C, Berthelet S, Krogan NJ, Wolek A, Podolny V, Carpenter LR, Greenblatt JF, Baetz K, Buratowski S. 2006. The *Saccharomyces cerevisiae* histone H2A variant Htz1 is acetylated by NuA4. Genes Dev 20:660–665. doi:10.1101/gad.1388106. 16543219 PMC1413285

[32] Kalocsay M, Hiller NJ, Jentsch S. 2009. Chromosome-wide Rad51 spreading and SUMO-H2A.Z-dependent chromosome fixation in response to a persistent DNA double-strand break. Mol Cell 33:335–343. doi:10.1016/j.molcel.2009.01.016. 19217407

[33] Larochelle M, Gaudreau L. 2003. H2A.Z has a function reminiscent of an activator required for preferential binding to intergenic DNA. EMBO J 22:4512–4522. doi:10.1093/emboj/cdg427. 12941702 PMC202369

[34] Jensen K, Santisteban MS, Urekar C, Smith MM. 2011. Histone H2A.Z acid patch residues required for deposition and function. Mol Genet Genomics 285:287–296. doi:10.1007/s00438-011-0604-5. 21359583 PMC3253533

[35] Wang AY, Aristizabal MJ, Ryan C, Krogan NJ, Kobor MS. 2011. Key functional regions in the histone variant H2A.Z C-terminal docking domain. Mol Cell Biol 31:3871–3884. doi:10.1128/MCB.05182-11. 21791612 PMC3165728

[36] Wratting D, Thistlethwaite A, Harris M, Zeef LAH, Millar CB. 2012. A conserved function for the H2A.Z C terminus. J Biol Chem 287:19148–19157. doi:10.1074/jbc.M111.317990. 22493515 PMC3365947

[37] Wood TJ, Thistlethwaite A, Harris MR, Lovell SC, Millar CB. 2013. Mutations in non-acid patch residues disrupt H2A.Z’s association with chromatin through multiple mechanisms. PLoS One 8:e76394. doi:10.1371/journal.pone.0076394. 24098487 PMC3788105

[38] Wu W-H, Alami S, Luk E, Wu C-H, Sen S, Mizuguchi G, Wei D, Wu C. 2005. Swc2 is a widely conserved H2AZ-binding module essential for ATP-dependent histone exchange. Nat Struct Mol Biol 12:1064–1071. doi:10.1038/nsmb1023. 16299513

[39] Halley JE, Kaplan T, Wang AY, Kobor MS, Rine J. 2010. Roles for H2A.Z and its acetylation in GAL1 transcription and gene induction, but not GAL1-transcriptional memory. PLoS Biol 8:e1000401. doi:10.1371/journal.pbio.1000401. 20582323 PMC2889906

[40] Coulombe C, Poitras C, Nordell-Markovits A, Brunelle M, Lavoie M-A, Robert F, Jacques P-É. 2014. VAP: a versatile aggregate profiler for efficient genome-wide data representation and discovery. Nucleic Acids Res 42:W485–493. doi:10.1093/nar/gku302. 24753414 PMC4086060

[41] Luk E, Ranjan A, Fitzgerald PC, Mizuguchi G, Huang Y, Wei D, Wu C. 2010. Stepwise histone replacement by SWR1 requires dual activation with histone H2A.Z and canonical nucleosome. Cell 143:725–736. doi:10.1016/j.cell.2010.10.019. 21111233 PMC7251641

[42] Dronamraju R, Ramachandran S, Jha DK, Adams AT, DiFiore JV, Parra MA, Dokholyan NV, Strahl BD. 2017. Redundant functions for Nap1 and Chz1 in H2A.Z deposition. Sci Rep 7:10791. doi:10.1038/s41598-017-11003-8. 28883625 PMC5589762

[43] Wang Y, Liu S, Sun L, Xu N, Shan S, Wu F, Liang X, Huang Y, Luk E, Wu C, Zhou Z. 2019. Structural insights into histone chaperone Chz1-mediated H2A.Z recognition and histone replacement. PLoS Biol 17:e3000277. doi:10.1371/journal.pbio.3000277. 31107867 PMC6544321

[44] Brewis HT, Wang AY, Gaub A, Lau JJ, Stirling PC, Kobor MS. 2021. What makes a histone variant a variant: changing H2A to become H2A.Z. PLoS Genet 17:e1009950. doi:10.1371/journal.pgen.1009950. 34871303 PMC8675926

[45] Sato S, Tanaka N, Arimura Y, Kujirai T, Kurumizaka H. 2020. The N-terminal and C-terminal halves of histone H2A.Z independently function in nucleosome positioning and stability. Genes Cells 25:538–546. doi:10.1111/gtc.12791. 32500630 PMC7496805

[46] Papamichos-Chronakis M, Watanabe S, Rando OJ, Peterson CL. 2011. Global regulation of H2A.Z localization by the INO80 chromatin-remodeling enzyme is essential for genome integrity. Cell 144:200–213. doi:10.1016/j.cell.2010.12.021. 21241891 PMC3035940

[47] Qiu H, Biernat E, Govind CK, Rawal Y, Chereji RV, Clark DJ, Hinnebusch AG. 2020. Chromatin remodeler Ino80C acts independently of H2A.Z to evict promoter nucleosomes and stimulate transcription of highly expressed genes in yeast. Nucleic Acids Res 48:8408–8430. doi:10.1093/nar/gkaa571. 32663283 PMC7470979

[48] Hardy S, Robert F. 2010. Random deposition of histone variants: a cellular mistake or a novel regulatory mechanism? Epigenetics 5:368–372. doi:10.4161/epi.5.5.11787. 20448466

[49] Tyagi M, Imam N, Verma K, Patel AK. 2016. Chromatin remodelers: we are the drivers!! Nucleus 7:388–404. doi:10.1080/19491034.2016.1211217. 27429206 PMC5039004

[50] Udugama M, Sabri A, Bartholomew B. 2011. The INO80 ATP-dependent chromatin remodeling complex is a nucleosome spacing factor. Mol Cell Biol 31:662–673. doi:10.1128/MCB.01035-10. 21135121 PMC3028646

[51] Krietenstein N, Wal M, Watanabe S, Park B, Peterson CL, Pugh BF, Korber P. 2016. Genomic nucleosome organization reconstituted with pure proteins. Cell 167:709–721.e12. doi:10.1016/j.cell.2016.09.045. 27768892 PMC5240917

[52] Brahma S, Udugama MI, Kim J, Hada A, Bhardwaj SK, Hailu SG, Lee T-H, Bartholomew B. 2017. INO80 exchanges H2A.Z for H2A by translocating on DNA proximal to histone dimers. Nat Commun 8:15616. doi:10.1038/ncomms15616. 28604691 PMC5472786

[53] Kumar Y, Bhargava P. 2013. A unique nucleosome arrangement, maintained actively by chromatin remodelers facilitates transcription of yeast tRNA genes. BMC Genomics 14:402. doi:10.1186/1471-2164-14-402. 23767421 PMC3698015

[54] Shimada K, Oma Y, Schleker T, Kugou K, Ohta K, Harata M, Gasser SM. 2008. Ino80 chromatin remodeling complex promotes recovery of stalled replication forks. Curr Biol 18:566–575. doi:10.1016/j.cub.2008.03.049. 18406137

[55] Ogiwara H, Enomoto T, Seki M. 2007. The INO80 chromatin remodeling complex functions in sister chromatid cohesion. Cell Cycle 6:1090–1095. doi:10.4161/cc.6.9.4130. 17471029

[56] Chambers AL, Ormerod G, Durley SC, Sing TL, Brown GW, Kent NA, Downs JA. 2012. The INO80 chromatin remodeling complex prevents polyploidy and maintains normal chromatin structure at centromeres. Genes Dev 26:2590–2603. doi:10.1101/gad.199976.112. 23207916 PMC3521627

[57] Jeronimo C, Robert F. 2014. Kin28 regulates the transient association of Mediator with core promoters. Nat Struct Mol Biol 21:449–455. doi:10.1038/nsmb.2810. 24704787 PMC3997488

[58] Pasquier E, Wellinger RJ. 2020. *In vivo* chromatin organization on native yeast telomeric regions is independent of a cis-telomere loopback conformation. Epigenetics Chromatin 13:23. doi:10.1186/s13072-020-00344-w. 32443982 PMC7243337

[59] Bourgey M, Dali R, Eveleigh R, Chen KC, Letourneau L, Fillon J, Michaud M, Caron M, Sandoval J, Lefebvre F, Leveque G, Mercier E, Bujold D, Marquis P, Van PT, Anderson de Lima Morais D, Tremblay J, Shao X, Henrion E, Gonzalez E, Quirion P-O, Caron B, Bourque G. 2019. GenPipes: an open-source framework for distributed and scalable genomic analyses. Gigascience 8:giz037. doi:10.1093/gigascience/giz037. 31185495 PMC6559338

[60] Bolger AM, Lohse M, Usadel B. 2014. Trimmomatic: a flexible trimmer for Illumina sequence data. Bioinformatics 30:2114–2120. doi:10.1093/bioinformatics/btu170. 24695404 PMC4103590

[61] Li H, Durbin R. 2009. Fast and accurate short read alignment with Burrows-Wheeler transform. Bioinformatics 25:1754–1760. doi:10.1093/bioinformatics/btp324. 19451168 PMC2705234

[62] Tarasov A, Vilella AJ, Cuppen E, Nijman IJ, Prins P. 2015. Sambamba: fast processing of NGS alignment formats. Bioinformatics 31:2032–2034. doi:10.1093/bioinformatics/btv098. 25697820 PMC4765878

[63] Heinz S, Benner C, Spann N, Bertolino E, Lin YC, Laslo P, Cheng JX, Murre C, Singh H, Glass CK. 2010. Simple combinations of lineage-determining transcription factors prime cis-regulatory elements required for macrophage and B cell identities. Mol Cell 38:576–589. doi:10.1016/j.molcel.2010.05.004. 20513432 PMC2898526

[64] Zhang Y, Liu T, Meyer CA, Eeckhoute J, Johnson DS, Bernstein BE, Nusbaum C, Myers RM, Brown M, Li W, Liu XS. 2008. Model-based analysis of ChIP-Seq (MACS). Genome Biol 9:R137. doi:10.1186/gb-2008-9-9-r137. 18798982 PMC2592715

