# Supplementary material for: The Histone Variant H2A.Z C-Terminal Domain Has Locus-Specific Differential Effects on H2A.Z Occupancy and Nucleosome Localization
Source: Microbiol Spectr. 2023 Feb 23;11(2):e02550-22. doi: 10.1128/spectrum.02550-22 (PMC10100702; doi:10.1128/spectrum.02550-22)
Supplement: Supplemental file 1 — Fig. S1 and S2. Download spectrum.02550-22-s0001.pdf, PDF file, 0.09 MB [file spectrum.02550-22-s0001.pdf]

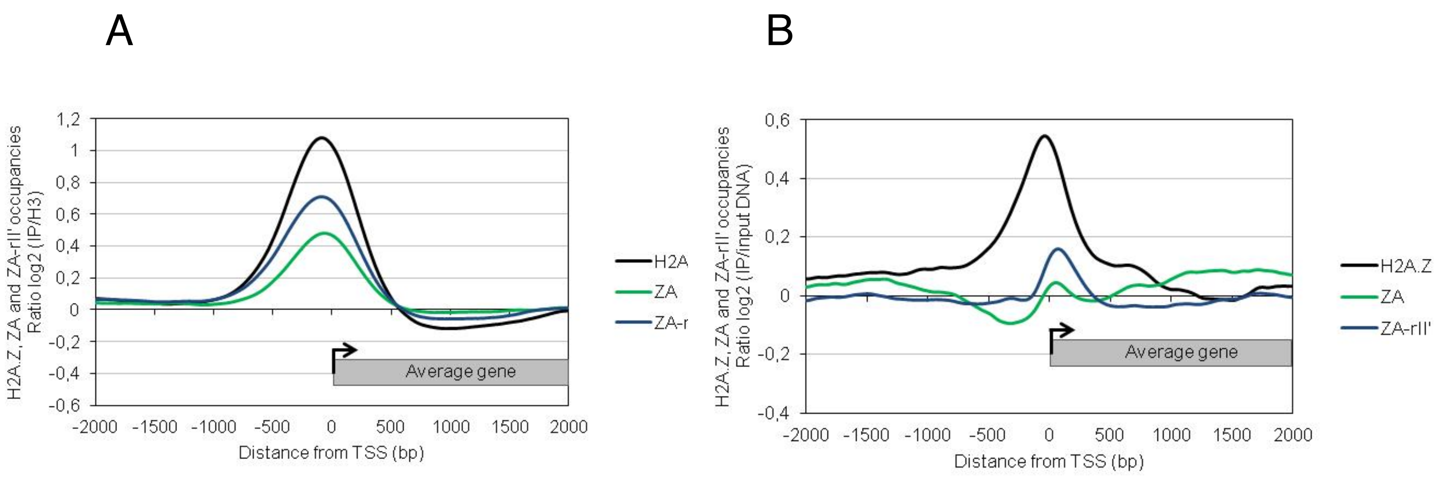

Supplementary Fig.1. Alignment of genomic data (smoothed H2A.Z occupancy as H2A.Z/H3 log_2 ratios **(A)** or H2A.Z/Input log_2 ratios **(B)** with respect to the TSS.

	**paired t-test p values compared to H2A.Z**	
	ZA	ZA-rII'
**CEN**	6.47490533681978E-88	1.00568046740193E-57
**ORI**	1.2885366878717E-59	6.35815029424028E-73
**HZAD**	1.42329080760869E-31	0.828447672078779
**tRNA**	1.315456862997E-72	7.6060606072191E-115
**snoRNA**	4.55104255545219E-53	3.87046833429579E-40

Supplementary Fig.2. p values of paired statistical t-tests of ZA and ZA-rII' association to various types of genomic loci, relative to WT H2A.Z.
